# Dispersive effects in imaging polarimetry based on a wire-grid polarizer

**DOI:** 10.1038/s41598-020-66512-w

**Published:** 2020-06-11

**Authors:** Seongmin Im, Gwiyeong Moon, Donghyun Kim

**Affiliations:** School of Electrical and Electronic Engineering, Yonsei University, Seoul, 03722 Korea

**Keywords:** Applied optics, Nanowires

## Abstract

We explore effects of light dispersion by a wire-grid polarizer (WGP) in imaging polarimetry. The dispersive characteristics of a WGP, combined with off-axis scene incidence, cause significant non-uniformity. The normalized performance measure of contrast due to dispersion of WGP exceeded 0.84 for transmittance and 0.90 for extinction ratio (maximum non-uniformity at 1 and 0 for uniform performance). Dispersion also produces a lateral spread in the imaging plane, which may induce spectral image misregistration. Without higher-order excitation, the misregistration can be at the least a few pixels long in the detector. In the presence of higher-order modes, the dispersive misregistration can be severe and critical for polarized scene extraction. The results emphasize the need for an imaging polarimeter to be designed to manage the dispersive effects.

## Introduction

Since 1960s, a wire-grid polarizer (WGP) has been considered as an optical element to discriminate polarization components:^1^ if an electric field of a light wave oscillates in parallel to the metallic wire-grids (TE polarization), it is largely absorbed, while it is transmitted if the electric field is perpendicular to the direction of wire-grids (TM polarization). Superb polarimetric performance combined with inherent structural planarity has made a WGP attractive in applications such as spectropolarimetry^2,3^ and projection displays^4^. Effects of a WGP were investigated in conjunction with a rotating platform^5^ and on the flexible^6–9^ and rough surface^10^. Thermal properties and plasmonic enhancement of a WGP were also studied^11–13^. On the other hand, a WGP was implemented using DNA-nanoparticle composites^14^. For the planarity of the structure, a WGP has been integrated to various electronic and photonic devices including a CMOS and CCD imaging sensor^15–19^, contact lens^20^, fiber-optics^21^, light-emitting diodes^22^, liquid crystal^23–26^, optical isolators^27,28^, a photodiode^29^, photoelectrochemical solar cells^30^, and semiconductor laser^31^.

Use of a WGP has been particularly beneficial for imaging polarimetry because complete polarization contents of a scene may be measured simply by changing the orientation of wire-grids on a pixelated platform in a flexible way^32–38^. Many design issues of WGPs have been explored in imaging polarimetry, for example, non-uniformity in the polarimetric performance depending on the incident scene vector^39^ and effect of finite pixel size^40^. WGP was integrated into a diffractive optical element^41^. One of the issues that have not been fully addressed and therefore we intend to address in this work is to understand the dispersive effects on the imaging performance which may appear in addition to typical spherical and chromatic aberration of imaging optics when WGPs are integrated for use in imaging polarimetry. Understanding the dispersive effects is critical to the design of WGPs because the polarimetry like any other imaging applications is performed in a broadband spectrum. More importantly, light dispersion can have an extremely severe and harmful effect on diffractive optical devices including metamaterials as recently reported for far-field applications^42,43^, which may limit practical application in imaging. In this sense, this study has a broad implication far greater than mere suggestion of a novel WGP design for imaging polarimetry.

## Model and method

### Numerical model for WGP

**Figure 1:**
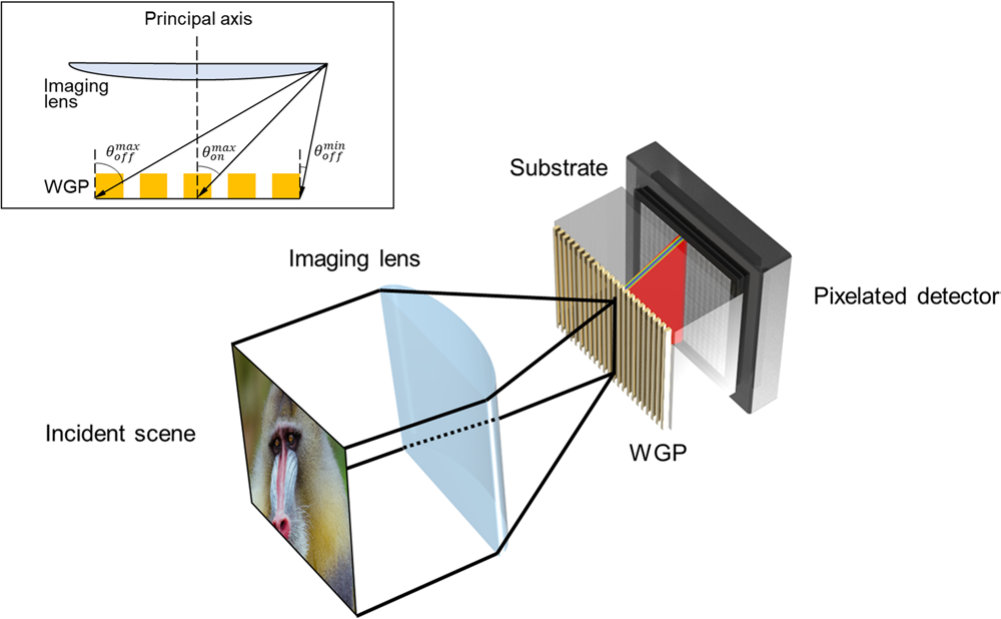
Schematic illustration of simplified imaging polarimetry based on a WGP. Incidence scene is imaged onto a pixelated detector through an imaging lens and a WGP mounted on a substrate. The scene undergoes dispersive effects of the WGP. The illustration only shows on-axis scene incidence for convenience. Inset figure represents off and on-axis scene incidence in cross-section. With respect to the principal axis, an on-axis scene is incident with an angle up to ${\theta }_{on}^{max}$, while the incident angle for an off-axis scene is between ${\theta }_{off}^{min}$ and ${\theta }_{off}^{max}$. Image of mandrill created by the US Department of State and available under the CC PDM 1.0 license.

Wire-grids were assumed to be made of gold on a BK7 substrate in air ambience, as shown in the schematic of Fig. 1. Additional optics that are typically used in imaging polarimetry have been omitted for simplification. Incident scene is illuminated in the visible waveband (λ = 400 ~ 700 nm). Refractive index of BK7 substrate (n_s_) and gold (n_m_) was obtained as n_s_ = 1.5308 and n_m_ = 1.4684 + j1.9530 for λ = 400 nm^44^. At λ = 700 nm, n_s_ = 1.5131 and n_m_ = 0.1310 + j4.0624. The fill factor *ff* is defined as the ratio of wire-grid width to the period *Λ*. Wire-grids are assumed to be rectangular in profile (width *ffΛ* and thickness *h*) and infinite along the y axis. Wire-grid periods were varied from *Λ* = 100 nm ~ 1 μm in a step of 100 nm for a fill factor *ff* = 0.10, 0.25, 0.50, 0.75, and 0.90. Thickness of wire-grids is fixed at 200 nm.

### Model for imaging polarimetry

In the imaging polarimetry model, a WGP is placed at the focal length (*f*) of an imaging convex lens (*f*-number *f/#*). For the convenience of analysis, we have assumed the imaging lens to be cylindrical, as shown in Fig. 1, with an incident angle for off and on-axis scene ranging from 0° to 60° as well as corresponding values of numerical aperture (NA) and *f*-number listed in Table 1. Spherical and chromatic aberration of the imaging lens has been disregarded in order to focus on the dispersive effects of a WGP. The aberration would further exacerbate the dispersive effects due to a WGP. The size of a WGP and an aperture, which is determined by the imaging lens, is denoted as *D*_*WGP*_ and *D*_*L*_. For generality, we have assumed that a WGP is not directly integrated into an imaging detector. For this consideration, the thickness of a WGP substrate is set to be *d*_*s*_ = 500 μm. The WGP-to-detector distance is *s*: *s* = 0 if the WGP operates in contact with the detector. A WGP and a detector are assumed to be aligned at the center in the lateral plane. The diameter of an Airy pattern (*D*_*Airy*_) produced by the aperture is given as:

1 $${D}_{Airy}\approx 2.44\lambda \frac{f}{D}=2.44\lambda (f/\#)=1.22\frac{\lambda }{{\rm{NA}}}=1.22\,\frac{\lambda }{n\,\sin \,\theta }.$$

*f* is the imaging focal length and *D* is the aperture diameter. *D*_*Airy*_ is listed in Table 1 with respect to *f*-#. As is implied in Eq. (1) and Table 1, use of a high NA imaging lens reduces the point-spread function, thus enhances imaging resolution, with a large angle of scene incidence. This, however, may be accompanied by adverse effects of higher-order diffraction. On the contrary, a lens with a small NA can avoid higher-order excitation, despite degradation of resolution.

**Table 1:** Airy diameter for λ = 400 and 700 nm (unit: μm), numerical aperture (NA), and maximum on-axis angle for scene incidence (${\theta }_{on}^{max}$), as the *f*-number of an imaging lens is varied.

*f*-#	Airy diameter (unit: μm)		NA	Angle of incidence
	λ = 400 nm	λ = 700 nm		
0.6	0.54	0.95	0.9	64°
0.7	0.65	1.14	0.75	48°
1	0.98	1.71	0.5	30°
1.25	1.22	2.14	0.4	23°
1.7	1.63	2.85	0.3	17°
2.5	2.44	4.27	0.2	11°
3.8	3.75	6.57	0.13	7°
5	4.88	8.54	0.1	5°

Generation of an object and image acquisition were performed on Matlab^TM^, i.e., spectral component images were generated in 512 × 512 pixels in a step of 10 nm from λ = 400 to 700 nm (total 31 images) by mapping RGB combinations in the CIE 1931 xy chromaticity space. Particle swarm optimization was used to find a specific single RGB combination. Each of the spectral component images was processed with *ER*(λ) between TE and TM polarization and combined to produce an image acquired by imaging polarimetry. Dispersive artifacts were then applied to the images.

### Numerical methods and performance metrics

We used rigorous coupled-wave analysis (RCWA) with 60 spatial harmonic orders to calculate optical characteristics of a WGP. For the assessment of polarimetric performance, transmittance (*T*) of TM and TE polarized light was measured in the far-field. Extinction ratio (*ER*) was used as a ratio of reflectance of TM polarization to that of TE polarization, i.e., *ER* = *T*_*TM*_/*T*_*TE*_. Out of the several factors contributing to non-uniform polarimetric performance of a WGP, we have focused on the angular off-axis and the dispersive effects, which we have quantified with normalized transmittance and extinction ratio. If we consider light rays making minimum and maximum incident angle, $\tan \,{\theta }_{on}^{min}$= 0° and $\tan \,{\theta }_{on}^{max}$= D_L_/2 *f* = 1/2 *f*/# for on-axis scene incidence. For off-axis incidence, $\tan \,{\theta }_{off}^{min}$= (1 ‒ D_WGP_/D_L_)/2 *f*/# and $\tan \,{\theta }_{off}^{max}$= (1 + D_WGP_/D_L_)/2 *f*/#. Insights on the performance non-uniformity due to off-axis imaging of the scene can be obtained by comparing *T* and *ER* at ${\theta }_{on}^{min}$ and ${\theta }_{off}^{max}$, respectively, as the best and the worst-case scenario. We have therefore defined off-axis non-uniformity in transmittance (*NT*_*off*_) and extinction ratio (*NER*_*off*_) as

2 $$N{T}_{off}=\frac{{T}_{TM}({\theta }_{off}^{max},\,\lambda )-{T}_{TM}({\theta }_{on}^{min},\,\lambda )}{{T}_{TM}({\theta }_{off}^{max},\,\lambda )+{T}_{TM}({\theta }_{on}^{min},\,\lambda )}$$

3 $$NE{R}_{off}=\frac{ER({\theta }_{off}^{max},\,\lambda )-ER({\theta }_{on}^{min},\,\lambda )}{ER({\theta }_{off}^{max},\,\lambda )+ER({\theta }_{on}^{min},\,\lambda )}.$$

Similarly, dispersive effects may be evaluated in the visible waveband by wavelength-dependent non-uniformity in transmittance (*NT*_*λ*_) and extinction ratio (*NER*_*λ*_) defined as

4 $$N{T}_{\lambda }=\frac{{T}_{TM}({\theta }_{off}^{max},\,\lambda =400\,nm)-{T}_{TM}({\theta }_{off}^{max},\,\lambda )}{{T}_{TM}({\theta }_{off}^{max},\,\lambda =400\,nm)+{T}_{TM}({\theta }_{off}^{max},\,\lambda )}$$

5 $$NE{R}_{\lambda }=\frac{ER({\theta }_{off}^{max},\,\lambda =400\,nm)-ER({\theta }_{off}^{max},\,\lambda )}{ER({\theta }_{off}^{max},\,\lambda =400\,nm)+ER({\theta }_{off}^{max},\,\lambda )}.$$

The non-uniformity ratios defined in Eqs. (2–5) becomes ±1 for the largest non-uniformity and approaches 0 for uniform performance. By the definition, *NT*_*λ*_ = *NER*_*λ*_ = 0 at λ = 400 nm.

## Results and discussion

### General characteristics based on the grating equation

By the conservation of momentum, grating equation specifies the direction of light propagation of diffractive optical devices in ambient and substrate modes, i.e.,

6 $$\sin \,{\theta }_{out}^{m}=\,\sin \,{\theta }_{in}+m\frac{\lambda }{\Lambda }$$

7 $$\sin \,{\theta }_{out}^{m}=\,\sin \,{\theta }_{in}+m\frac{\lambda }{{n}_{s}(\lambda )\Lambda }.$$

Here, *m* is an integer representing the mode number. *θ*_*in*_ and *θ*_*out*_^*m*^ denote the angle of light incidence and an outgoing wave corresponding to the *m*-th mode. *θ*_*in*_ is not a function of λ under the assumption of no aberration of the imaging lens. The results suggest excitation of higher-order modes at normal incidence for λ ≥ 264 nm. For inclined light incidence, the wire-grid period *Λ* at which higher-order diffraction kicks in becomes even shorter.

**Figure 2:**
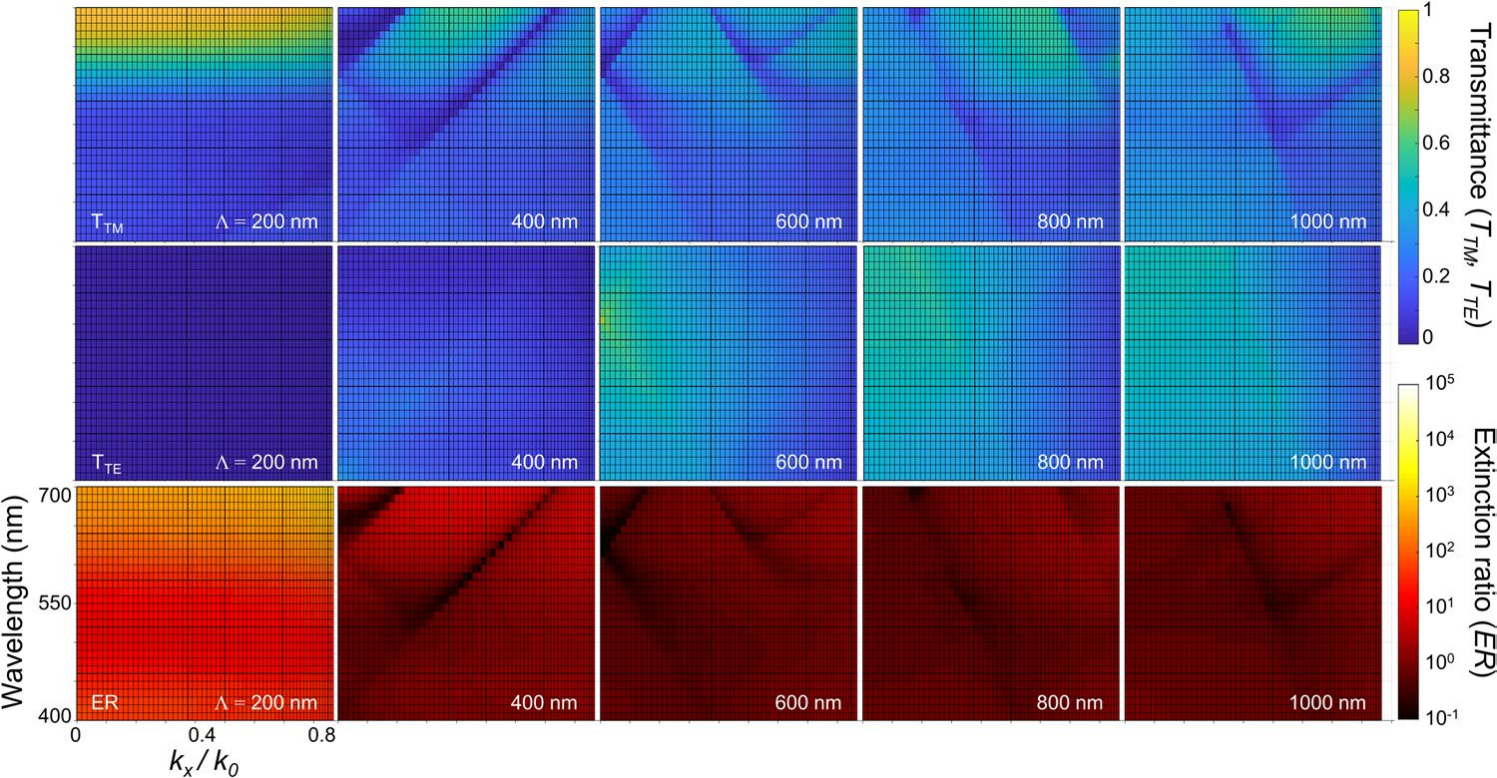
*T*_*TM*_, *T*_*TE*_, and *ER* for wire-grid period *Λ* = 200, 400, 600, 800, and 1000 nm. *k*_*x*_/*k*_0_ = sin(*θ*_*in*_) in the *x*-axis (*k*_0_: free-space wave number). Wavelength λ in the *y*-axis was varied in 400 ~ 700 nm. It was assumed that *ff* = 0.50 and height of 200 nm. Higher-order diffraction components are shown for *Λ* > 200 nm.

Figure 2 presents TTM, TTE, and ER, which in effect shows dispersion relations between momentum in the x-axis and energy in the *y*-axis. The momentum is normalized by the free-space wave number *k*_0_ = 2π/λ. A few notes are worth a mention. First, higher-order modes appear as light momentum is increased with a larger angle of incidence. Higher-order modes also become more evident with a longer wire-grid period. Such a trend is particularly strong for TM polarization in *T*_*TM*_ and *ER*. With TE polarized light, while higher-order modes also exist, overall transmittance is too much suppressed for the higher-order modes to emerge visibly.

**Figure 3:**
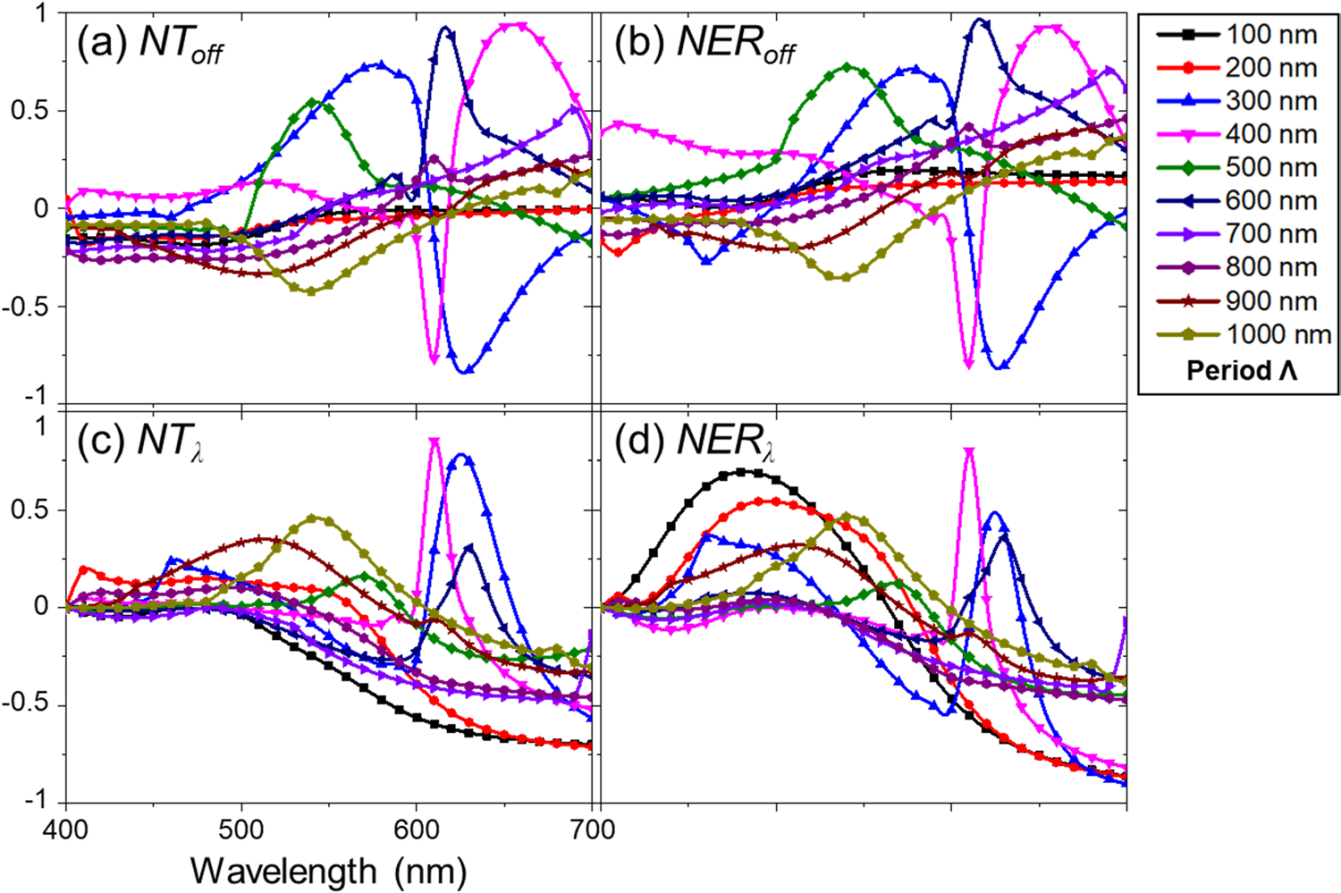
Non-uniformity ratios regarding the effects of off-axis scene components: (**a**) *NT*_*off*_ and (**b**) *NER*_*off*_. Dispersive effects presented by (**c)** *NT*_*λ*_ and (**d**) *NER*_*λ*_. Data were collected at every 10 nm of wavelengths, i.e., *Δλ* = 10 nm, for WGPs with *ff* = 0.50 and height of 200 nm. For other wavelength points, the data were interpolated by cubic splines. Off-axis and wavelength-dependent non-uniformity ratios are calculated for various wire-grid grating periods (*Λ*) of a WGP between *Λ* = 100 and 1000 nm (see the legend).

Effect of off-axis scene components in WGP-based imaging polarimetry is presented in Fig. 3(a,b). Because polarimetric performance of a WGP depends on light incidence, the presence of off-axis scenes creates significant non-uniformity in the performance, as described earlier^39^. Figure 3(a,b) address the dynamics due to scene incidence with an angular spread given by the finite NA. The results suggest that the deviation in the imaging plane can be extremely high: in terms of *NT*_*off*_ and *NER*_*off*_, the non-uniformity is shown to reach up to |*NT*_*off*_ | _max_ = 0.93211 at *Λ* = 400 nm and |*NER*_*off*_ | _max_ = 0.93624 at *Λ* = 600 nm. In other words, a scene component at a specific angle of incidence may dominate or be suppressed in an image acquired after a WGP.

### Dispersive effects on non-uniformity and image misregistration

We now address effects of wavelength-dependent dispersion. Dispersive effects may be manifested in two ways that are potentially inter-related. First, dispersion may also cause non-uniformity in the polarimetric performance that is additional to the effect of off-axis scene components, as shown in Fig. 3(c,d), i.e., Fig. 3(c,d) address spectral performance variation in reference to λ = 400 nm to measure how far the performance quantities diverge as the wavelength becomes longer in the visible waveband for the range of wire-grid period *Λ* = 100 ~ 1000 nm. Although dispersive non-uniformity appears to be less significant than that caused by angle-dependent off-axis light incidence, it is still quite high, i.e., |*NT*_*λ*_ | _max_ = 0.84935 at *Λ* = 400 nm and |*NER*_*λ*_ | _max_ = 0.90139 at *Λ* = 300 nm. This suggests that specific color components in a scene may be more emphasized in imaging polarimetry, depending on the structure of a WGP. Figure 3 also shows that the non-uniformity associated with dispersion of WGP is slightly less severe than that due to off-axis scene components, e.g., |*NT*_*λ*_ | _max_/|*NT*_*off*_ | _max_ = 0.91 if compared based on maxima. Interestingly, dispersive non-uniformity observed in Fig. 3(c,d) is quite prominent at a short wire-grid period, i.e., *NT*_*λ*_ and *NER*_*λ*_ can be significantly large at *Λ* = 100 nm. In other words, imaging polarimetry using a WGP with extremely fine wires, while it may minimize off-axis non-uniformity, may not be able to avoid dispersive non-uniformity in the performance.

**Figure 4:**
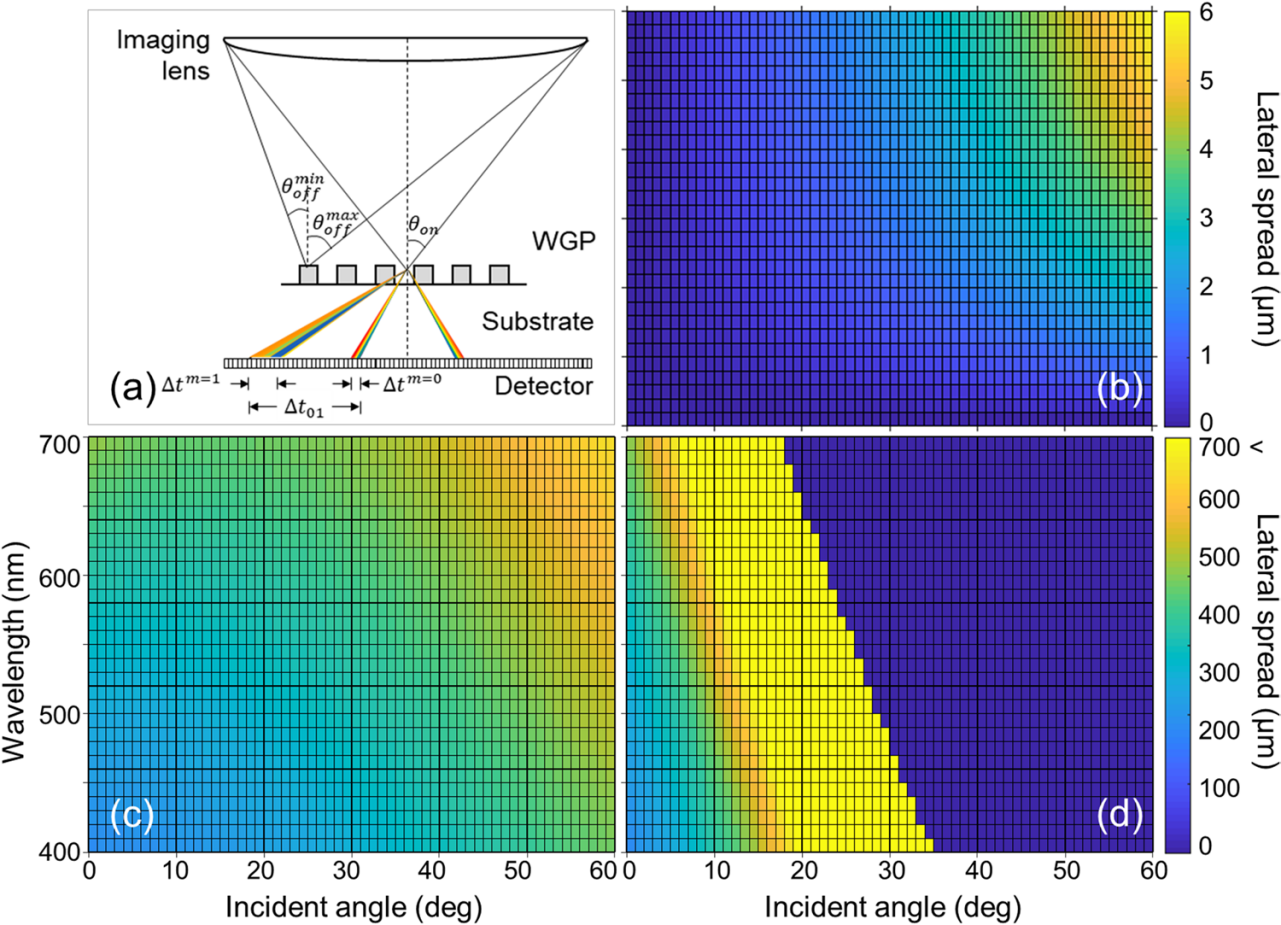
(**a**) Schematic illustration of higher-order excitation in imaging polarimetry. The illustration shows dispersive lateral spread: $\Delta {{\boldsymbol{t}}}^{{\boldsymbol{m}}=0}$ and $\Delta {{\boldsymbol{t}}}^{{\boldsymbol{m}}=1}$ for the zeroth and +1^st^-order mode. $\Delta {{\boldsymbol{t}}}_{01}$ for the overall dispersive spread. For convenience, lateral spreads are only shown for on-axis light incidence. (**b**) Dispersive lateral spread $\Delta {{\boldsymbol{t}}}^{{\boldsymbol{m}}=0}$ without higher-order modes. Calculated dispersive spread: (**c**) −1^st^ and (**d**) +1^st^-order. Wavelength was sampled at every 10 nm in the range of 400–700 nm. The lateral spread was obtained for a WGP with *ff* = 0.50, period *Λ* = 1000 nm, and height of 200 nm.

Secondly, dispersive effects may produce image misregistration in the imaging plane. Schematic illustration is provided in Fig. 4(a). For simplicity, we consider zeroth and higher-order effect separately. The zeroth-order effect of dispersion can be understood by the Snell’s law, *i.e*.,

8 $${n}_{s}(\lambda )\sin \,{({\theta }_{out}^{m=0})}_{12}=\,\sin \,{\theta }_{in}.$$

Equation (8) describes light refraction into the glass substrate, where subscript ()_12_ denotes propagation from air ambience into glass substrate. Upon the second refraction at the bottom surface, the propagation angle of the outgoing wave becomes identical to that of an incident light, i.e., ${\theta }_{out}^{m=0}={\theta }_{in}$. Because of dispersion, a lateral spread arises in proportion to the thickness of a WGP substrate, which serves as a measure of image misregistration among various spectral components. Assuming that an imaging detector is in contact with a WGP, the refracted light becomes spectrally spread with a spread

9 $$\Delta {t}^{m=0}=t|\tan \,{\theta }_{out}^{m=0}(\lambda )-\,\tan \,{\theta }_{out}^{m=0}(\lambda =400\,nm)|.$$

Figure 4(b) presents $\Delta {t}^{m=0}$ with respect to wavelength λ and incident angle *θ*_*in*_. Assuming no higher-order diffraction, and with a typical size of a pixel of an imaging detector on the order of 10 ~ 100 μm, it is suggested that $\Delta {t}^{m=0}$ should not cause a significant problem. However, potential imaging misregistration between images corresponding to various spectral components may arise at the edges of a pixel. This issue would particularly be critical, considering recent emergence of imaging detectors with extremely small pixels, e.g., a CCD image sensor with submicron pixels was reported^45,46^. With a pixel of 1-μm size, the results in Fig. 4(b) suggest misregistration on the order of six pixels.

Once higher-order modes are excited, they serve as noise for the most part. For example, it is well-known that higher-order modes suffer from relatively low polarimetric extinction. In this work, for the higher-order modes, we have defined the lateral spread in the worst case as

10 $$\Delta {t}_{0m}=t|\tan \,{\theta }_{out}^{m}(\lambda )-\,\tan \,{\theta }_{out}^{m=0}(\lambda =400\,nm)|.$$

which includes higher-order diffraction in addition to the effect of chromatic dispersion. For this reason, we emphasize that the overall dispersive misregistration can be greatly amplified for the higher-order modes and the deviation be potentially significant depending on the orders, as clearly shown in Fig. 4(c,d). Therefore, the result implies requirement of higher-order diffraction components to be either fully filtered or suppressed using WGPs with a sufficiently short *Λ*.

### Dispersive effects in Stokes polarimetry

**Figure 5:**
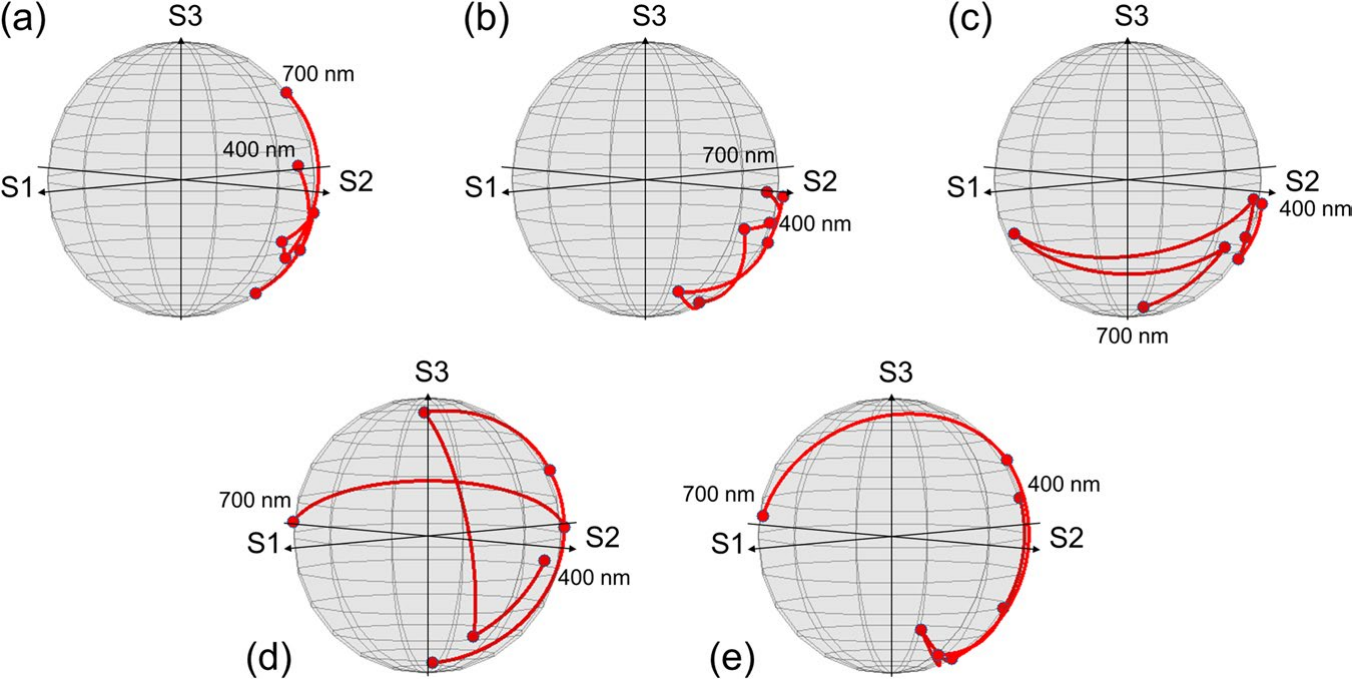
Variation of polarization states after transmission through a WGP is shown in a red line on the Poincaré sphere for (**a**) *θ*_*in*_ = 0°, (**b**) 15°, (**c**) 30°, (**d**) 45°, and (**e**) 60°. Incident light wavelength ranges from *λ* = 400 to 700 nm with a 50-nm step in the visible waveband.

Before we present diattenuation-based dispersive effects in imaging polarimetry, it is also of interest to explore the effect on more general Stokes polarimetry. For convenience, we assume single-point acquisition in the far-field with unpolarized light incidence. The results are shown in Fig. 5 where changes of polarization states are described on the Poincaré sphere when the angle of incidence (*θ*_*in*_) as well as the wavelength (*λ*) is varied from *θ*_*in*_ = 0 ~ 60° in the visible waveband.

It is quite clear that the polarization states may vary drastically due to light dispersion. Interestingly, the spectral variation of polarization tends to increase with a high angle of incidence due both to the difference in phase change that TE or TM polarization undergoes with respect to the incidence angle and also to the changes in the relative amount between the components polarized in parallel and orthogonal to the wire-grids^47–49^. If we quantify the variation of polarization states with a sum of arc lengths (*L*_*p*_) measured between points on the Poincaré sphere (initially from *U* = 1), d*L*_p_/d*θ*_*in*_ = 0.5795 and d*L*_p_/d*λ* = 1.185. More importantly, we emphasize that the polarization state varies with light wavelength and may thus be affected significantly by the spectral content of an incident scene. The scene-based variation of polarization states may be additive to the dispersive effects that were presented previously. For more explicit understanding of dispersive effects in imaging polarimetry, however, we only consider diattenuation properties of a WGP in the following section.

### Imaging polarimetry with dispersive effects

**Figure 6:**
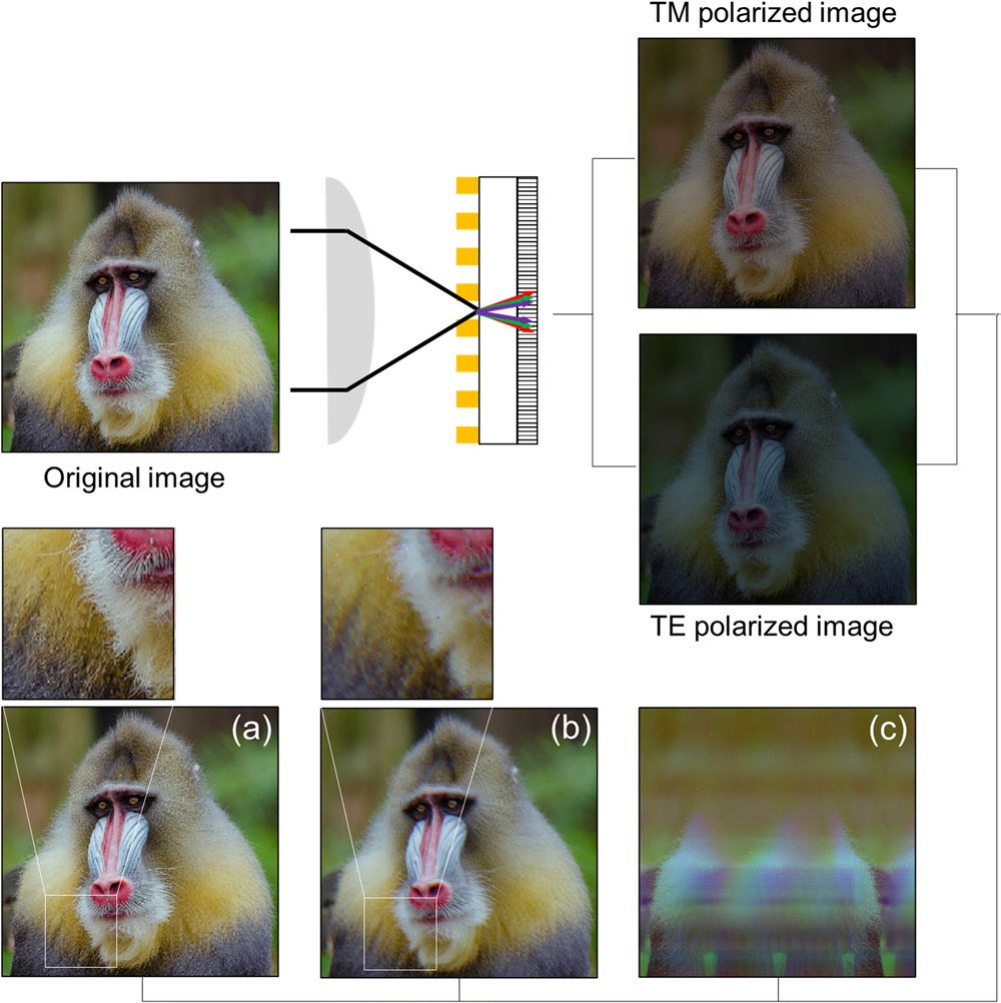
Dispersive effects simulated on images acquired in imaging polarimetry. (**a**) Ideal image without dispersion. Images that suffer from (**b**) zeroth-order and (**c**) 1^st^-order dispersion. Insets demonstrate loss of clarity as a result of dispersive effects in (**b**) vs. no dispersion in (**a**). Image of mandrill created by the US Department of State and available under the CC PDM 1.0 license.

Under the conditions without dispersive artifacts, polarimetric performance is represented by *ER*. Now, performance non-uniformity and image misregistration due to light dispersion degrades overall polarimetric content in a pixel. Figure 6 presents images with and without dispersive artifacts, i.e., an image was initially converted into an object by mapping RGB combinations in the CIE 1931 xy chromaticity space. Here, we have used a baboon image, which is one of the test images often employed in the image processing community. Because the lateral spread due to wavelength dispersion is exacerbated in the presence of higher-order diffraction, the worst-case performance occurs at a large angle of incidence with a long wire-grid period (*Λ*) greater than light wavelength. For the simulation of dispersive effects, therefore, the wire-grid period of WGP and incident angle were assumed to be *Λ* = 1000 nm and *θ*_*in*_ = 60° as the worst case. The baboon image as an object was acquired through a WGP as 50:50 TM and TE component. A TM image was obtained as transmitted through a WGP, while TE image intensity was proportionately reduced with *ER*(λ). The TM and TE component images were then combined to form a final ideal image without dispersion in Fig. 6(a). This is compared with an image in the presence of dispersive effects corresponding to the zeroth-order and also the case including higher-order diffraction modes, respectively shown in Fig. 6(b,c). For the visual convenience, higher-order images were normalized by the peak intensity of the (−1)^st^ TM image. In this illustration of an example, the detector sensitivity was assumed to be independent of wavelengths. With wavelength dependence, the contrast between ideal and dispersive images can be even starker.

The comparison of the magnified images laden with dispersive artifacts in the inset of Fig. 6(b) to that of an ideal image presented in Fig. 6(a) emphasizes that the dispersion induced by a WGP degrades the quality of images acquired in imaging polarimetry and the management of dispersion can therefore be crucial to the performance. Although we have assumed a simple WGP with wire-grids aligned in a single direction, a pixelated WGP with multiple orientations would incur dispersive effects much more complicated than are presented in Fig. 6. It is thus suggested that the results should be reflected in the design principles of imaging polarimetry and can be critical in applications of a WGP that operates in a broadband spectrum at a large angle of incidence using an imaging lens with a high NA, and when the pixel of an imaging detector is small comparable with the diffraction limit^50–52^. For example, higher-order diffraction modes need to be strictly suppressed, e.g., using a WGP with sufficiently fine wire-grids or employing filters in the light paths. To remove or reduce zeroth-order dispersive effects, a WGP is best integrated directly with a detector or in the closest proximity. A WGP mounted in an optical plane that is relayed in a 4 *f* imaging system to an image plane would cause more severe artifacts because of the distance between a WGP and a detector. This may be compensated using dispersive devices, e.g., gratings, in the optical path.

## Concluding remarks

In summary, we have investigated effects of dispersion in imaging polarimetry based on WGPs. Mainly, there are mainly two effects: dispersion aggravates performance non-uniformity, which is less severe than one that is caused by off-axis imaging. In addition, dispersive lateral spread produces image misregistration in the detector. The spread is expected to be smaller than or on the order of 10 μm in typical imaging conditions. This may be significantly worse when higher-order diffraction components are excited and can add systemic noise to the acquired images. The results therefore emphasize the need to consider dispersive artifacts for the design of WGP-based imaging polarimetry.
